# Funnel-Shaped Floating Vessel Oil Skimmer with Joule Heating Sorption Functionality

**DOI:** 10.3390/polym14112269

**Published:** 2022-06-02

**Authors:** Blake Herren, Mrinal C. Saha, M. Cengiz Altan, Yingtao Liu

**Affiliations:** School of Aerospace and Mechanical Engineering, University of Oklahoma, Norman, OK 73019, USA; blake.herren@ou.edu (B.H.); msaha@ou.edu (M.C.S.); altan@ou.edu (M.C.A.)

**Keywords:** oil/water separation, PDMS sponge, Joule heating, nanocomposite, oil skimmer

## Abstract

Floating vessel-type oil collecting devices based on sorbent materials present potential solutions to oil spill cleanup that require a massive amount of sorbent material and manual labor. Additionally, continuous oil extraction from these devices presents opportunities for highly energy-efficient oil skimmers that use gravity as the oil/water separation mechanism. Herein, a sorbent-based oil skimmer (SOS) is developed with a novel funnel-shaped sorbent and vessel design for efficient and continuous extraction of various oils from the water surface. A carbon black (CB) embedded polydimethylsiloxane (PDMS) sponge material is characterized and used as the sorbent in the SOS. The nanocomposite sponge formulation is optimized for high reusability, hydrophobicity, and rapid oil absorption. Joule heating functionality of the sponge is also explored to rapidly absorb highly viscous oils that are a significant challenge for oil spill cleanup. The optimized sponge material with the highest porosity and 15 wt% CB loading is tested in the SOS for large-scale oil spill extraction tests and shows effective cleaning of oil spilled on the water surface. The SOS demonstrates a high maximum extraction rate of 200 mL/min for gasoline and maintains a high extraction rate performance upon reuse when the sponge funnel is cleaned and dried.

## 1. Introduction

Due to the massive amount of exploration, storage, and transportation of oils and organic solvents, accidental spills in bodies of water remain a global challenge. While the yearly number of oil spills has reduced significantly in recent years [1], the technologies used to clean up oil spills have not significantly improved. Chemical dispersants and in-situ burning methods have been widely used to manage oil spills; however, these methods have contributed to additional environmental problems and increased health risks for the workers involved [2,3]. Mechanical methods used for removing oil spills from the water include booms to corral the oil, skimmers to extract the congregated oil slicks, and sorbent materials to soak up thin oil sheens. To extract oil from water, traditional oil skimmers require energy input to rotate a typically oleophilic media (belts, discs, drums, tubes, etc.) through the oil layer to transfer the oil to a collection chamber where it can be extracted with a pump. This energy-consuming oil/water separation mechanism makes traditional oil skimmers difficult to deploy and scale up, and they are highly inefficient for extracting thin oil layers from water [4]. Sorbent materials in sheet form are often deployed by hand to absorb and separate thin oil sheens from water. Most sorbents used in the industry today are single-use and account for significant solid waste that often far outweighs the liquid waste of the oil spill itself, with ratios as high as 400:1 [5]. This problem has made reusable and high sorption capacity sorbent materials a prominent field of research today. 

Several researchers in the field have focused on modifying the surface of highly porous substrates to improve the hydrophobic and oleophilic properties of sorbent materials [6]. Desired characteristics of state-of-the-art oil/water separation materials include lightweight with high oil sorption capacities, super-hydrophobicity, durability, reusability, low cost, and facile fabrication [7]. Advanced sorbent materials have also been developed to include unique functionalities including resistance to ultrahigh temperatures for in-situ oil burn-off from the sorbent [8], magnetism to facilitate sorbent recovery via magnets [9,10], and stimuli-responses such as resistive heating (Joule heating) and photothermal effect for viscous oil absorption [11,12,13]. Joule heating a semiconductive sorbent material is arguably the most applicable and versatile of these advanced sorbent features to heat and lower the viscosity of nearby oils to facilitate rapid absorption of highly viscous pollutants. However, little work has been done to scale up these state-of-the-art sorbent materials by utilizing low-cost materials and facile fabrication methods. 

The most promising sorbent-based technologies to reduce the amount of manual labor required and solid waste produced from oil spill cleanup include oil collecting vessels and oil extraction devices [7]. The goal of these devices is to utilize the properties of the sorbent materials to collect the oil in a container or continuously extract the oil. The simplest of these devices includes placing a tube into the center of a sorbent, such as an oleophilic and hydrophobic sponge, and pumping to continuously extract the oil through the sorbent [14,15,16]. However, the oil extraction rate is extremely slow using this technique and often results in some water extracted through the sponge [17]. Vessel-type oil collectors utilize a sorbent material and gravity (or an external force) to fill an oil collection chamber or vessel [18,19,20,21]. A few studies have demonstrated the ability to remove oil collected in these sorbent-based devices with a pump for brief small-scale demonstrations [22,23,24]. However, to the best of the authors’ knowledge, no previous study has developed a fully reusable floating vessel-type device with continuous oil extraction capabilities. 

In this study, for the first time, a floating vessel-type oil collector capable of continuous oil extraction from the water surface was developed. The novel sorbent-based oil skimmer (SOS) was created and tested to demonstrate continuous oil extraction from water, facile deployment, and high reusability. The gravity-driven oil/water separation mechanism of the novel oil skimmer required no energy input. The material properties of the nanocomposite sponges with varying porosities and carbon black (CB) loadings (Table S1, Supporting Information) were investigated including Joule heating, durability, hydrophobicity, and oleophilic properties. The reusability of the SOS was tested, various sponge funnel sheet designs were explored, and the ability of the SOS to extract different types of oil from fish tank-scale oil spill simulations was demonstrated. 

## 2. Materials and Methods

### 2.1. Materials

Sylgard 184 polydimethylsiloxane (PDMS) was obtained from Dow (Midland, TX, USA). Sodium chloride (NaCl) salt, gasoline, and diesel fuel were acquired from Walmart (Bentonville, AR, USA). The motor oil (30W-50) and polylactic acid (PLA) filament was purchased from Amazon (Seattle, WA, USA). Carbon black (CB), EPON 862 epoxy, Epikure 9553 curing agent, ethanol, tetrahydrofuran (THF), chloroform, acetone, silicone oil, and pump oil were bought from Sigma Aldrich (St. Luis, MO, USA). 

### 2.2. Sponge Fabrication

First, Part A (resin) and Part B (curing agent) of Sylgard 184 polymer were mixed at the weight ratio of 10:1 and were reinforced by CB via centrifugal mixing to create a nanocomposite prepolymer (CB/PDMS). The CB/PDMS was hand mixed with salt to create a mixture (PCS). The PCS with various salt and CB loadings (Table S1) was molded with 3D printed templates (Figure S1) into small (1 cm × 1 cm × 1 cm) and large (5 cm × 5 cm × 5 cm) cubes. The samples were cured, submerged in water to remove the salt porogen, cleaned with ethanol, and dried.

### 2.3. Characterization

A light microscope was used to image waterdrop contact angle tests and Joule heating motor oil absorption experiments by depositing a droplet (~10 μL) onto a sponge. The contact angles were measured using the software ImageJ from the recorded optical images. The porosity of the manufactured porous nanocomposites was measured using the density method and calculated using the Equation (1) below:

(1) $$\mathrm{Porosity}\;=\left[1-\frac{\rho_{p}}{\rho_{s}}\right]\times 100\%$$

where $\rho_{p}$ is the density of porous samples calculated by measuring the weight and volume of porous nanocomposites, and $\rho_{s}$ is the density of porous samples calculated by measuring the weight and volume of solid nanocomposites.

A scanning electron microscope (Zeiss NEON, Oberkochen, Germany) was used to image the surface of the various sponges. Sponge cubes were placed in various oils and organic solvents and were measured and weighed before and after absorption. The mass sorption capacity (C) was calculated following Equation (2).

(2) $$C=\frac{\left(M-\;m\right)}{m}$$

where M is the weight of the sponge after oil absorption and m is the dry weight of the sponge. The swelling ratio (S) was calculated with Equation (3).

(3) $$S=\frac{V}{v}$$

where V is the volume of the sponge after oil sorption and v is the volume of the dry sponge. All mechanical compression tests were performed on a single-column mechanical testing machine (Instron 3345, Norwood, MA, USA). A Viscometer (Brookfield DV-II+, Middleboro, MA, USA) was used to measure the viscosity of the motor oil at various temperatures. Silver epoxy and copper tape were applied on sponges as electrodes for Joule heating experiments measured with a digital thermometer (Fluke 54 II, Everett, WA, USA) and to measure the resistances of the small sponges with a resistance meter (Hioki RM3545-02, Plano, TX, USA) (Figure S2).

### 2.4. SOS Construction

The SOS prototype vessel was 3D printed in PLA and coated with several layers of epoxy. High-density polyethylene (HDPE) lid extensions were bolted onto the vessel. The sponge funnel was fabricated using 3D printed templates. First, a 1 cm thick circular sheet was formed by pressing the uncured PCS into a 3D printed template and the vent hole was cut out of the center of the sheet. A large extended cone-shaped salt porogen was fabricated by mixing salt with water, molding, and microwaving to harden (Figure S3). The porogen was placed on the PCS sheet over the top of a large hole in the center and covered with PCS, leaving a small 5 cm diameter hole at the tip of the large cone-shaped porogen. The sponge funnel was cured and placed in water to remove the salt porogen, cleaned with ethanol, and dried. To assemble the SOS, the sponge funnel was placed into the vessel and the lid and vent component was placed on top with the vent through the vent hole on the sponge funnel. The top HDPE sheet was placed on top of the lid and tied to the HDPE sheets bolted onto the vessel on both ends. Rope handles were added to facilitate SOS deployment and oil tubing was connected underneath the vessel.

### 2.5. SOS Testing

The SOS prototype with a CB15P9 sponge funnel was deployed in several 30 mm thick simulated oil spills to measure the oil extraction rate at each oil layer thickness. The sponge funnel designs tested: (i) small-diameter circular sponge sheet (diameter = 21 cm), (ii) large-diameter circular sponge sheet (diameter = 31 cm), and (iii) large-diameter flower-shaped sponge sheet (diameter = 31 cm; 60 pedals). The reusability of a sponge funnel with a small sponge sheet was determined by repeating extraction tests of gasoline with relevant sponge funnel reuse conditions (Supplementary Material Movie S1). Lastly, the SOS with the large-diameter sponge sheet was tested in a diesel fuel and crude oil simulated oil spill. 

## 3. Results and Discussion

### 3.1. SOS Inspiration, Design, and Functionality

The continuous oil extraction capabilities of the SOS were enabled by three key features: (i) the sponge funnel geometry, (ii) the oil sorbent material properties, and (iii) the vessel. The SOS design was conceptualized after preliminary experiments focused on scaling up polydimethylsiloxane (PDMS) sponges that have been limited to small-scale experiments largely due to lab-scale fabrication methods widely used, including the sugar cube templating method [25]. In this study, a simple PDMS, carbon black (CB), and salt mixture (PCS) was used to fabricate sorbent sponges that allowed for facile molding of several complex geometries. The PCS was used to encase a large spherical salt porogen to create a hollow sponge or shell structure (Figure S4). During deployment of the spherical sponge shell in a small-scale simulated oil spill, the inner core of the shell structure below the oil layer filled with oil due to gravity (Figure S5). Next, a pump was connected underneath the spherical sponge shell to extract the oil from the hollow sorbent (Figure S6). While this approach was novel, the sponge shell collapsed during extraction, demonstrated a slow absorption rate due to a small surface area of sorbent in contact with the thin oil layer, and struggled to maintain vertical alignment. To solve these issues, the sponge funnel was designed and fabricated to include a hollow funnel-shaped sponge shell connected to a large sponge sheet with a vent hole for high surface area contact with the oil layer (Figure S7). A vessel was incorporated with the sponge funnel to maintain vertical alignment and encase the separated oil to enable rapid oil extraction with a peristaltic pump connected to the vessel underneath the sponge funnel. 

**Figure 1:**
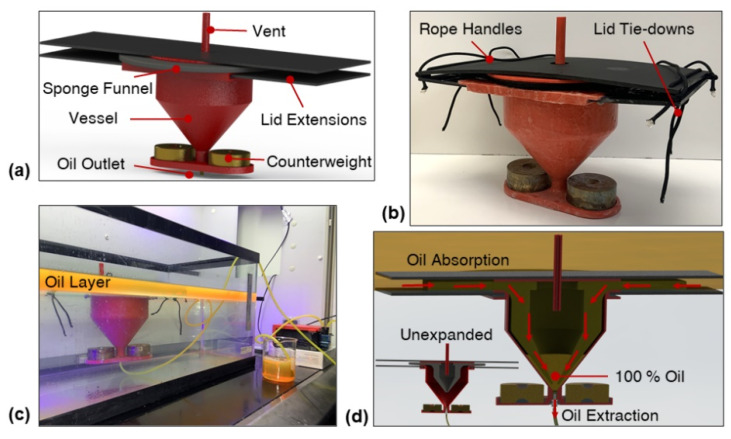

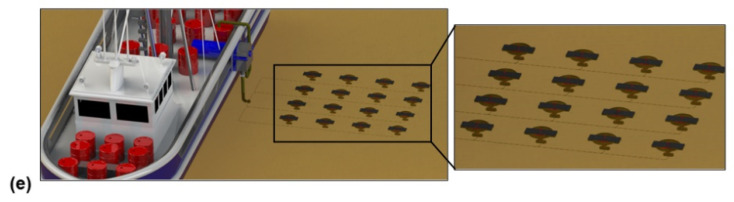
(**a**) The SOS design, pictures of (**b**) the fully constructed SOS prototype and (**c**) the SOS continuously extracting gasoline from the water surface, (**d**) a schematic of gravity-driven oil extraction functionality of the SOS, and (**e**) a conceptual image of an SOS array connected to one peristaltic pump.

The SOS prototype design used in this study included only counterweights; however, a ballast tank may be used to control the buoyancy of the device (Figure S8). The SOS prototype design (Figure 1a) utilized lid extensions due to the limited build volume of the 3D printer used to fabricate the vessel and to account for the expansion of the sponge funnel during oil absorption. The lid was tied down securely to create a leakproof seal between the oil sorbent and vessel (Figure 1b) such that no water could leak into the vessel with a sufficiently hydrophobic sponge funnel during oil extraction tests (Figure 1c). The gravity-driven oil/water separation mechanism of the novel oil skimmer (Figure 1d) required no energy input, which may allow for an array of SOS devices connected to one peristaltic pump for highly energy-efficient and rapid extraction of large-scale oil spills (Figure 1e). 

### 3.2. Development and Characterization of Sorbent Material

**Figure 2:**
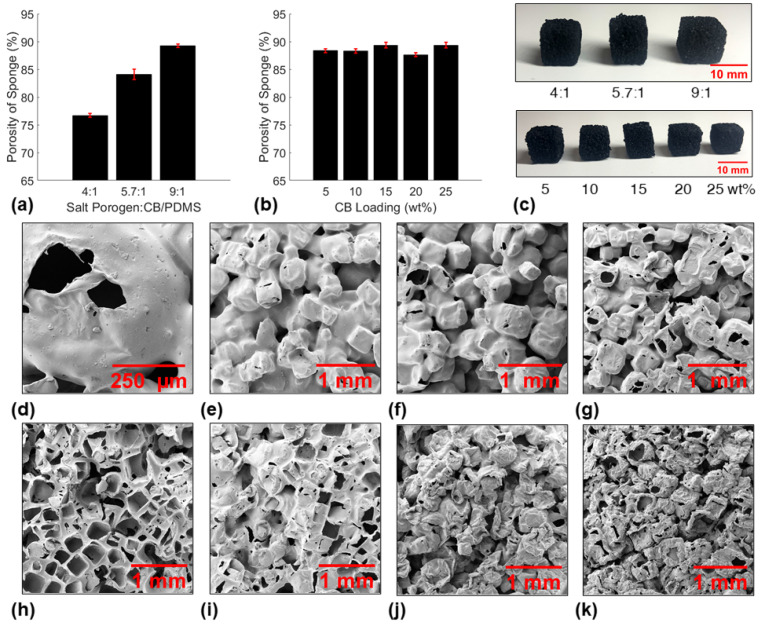
Porosities of sponges fabricated with varying (**a**) salt porogen:CB/PDMS prepolymer ratios and (**b**) CB loadings. (**c**) Pictures of the small sponge cubes fabricated for sorbent material characterization (top: CB15P4, CB15P6, and CB15P9; bottom: CB5P9, CB10P9, CB15P9, CB20P9, CB25P9). SEM images of (**d**) pores in a CB5P9 sponge and the microstructures of (**e**) CB15P4, (**f**) CB15P6, (**g**) CB15P9, (**h**) CB5P9, (**i**) CB10P9, (**j**) CB20P9, and (**k**) CB25P9 sponges.

The PCS mixture was used to fabricate sponge cubes consisting of CB dispersed within a PDMS matrix for material characterization. Several studies in the field attached nanoparticles to the surface of highly porous sponges to impart superhydrophobic or multi-functional properties like magnetism [26,27,28]. However, van der Waals forces between nanofillers and PDMS are relatively weak [29], and dip-coating and other nanoparticle attachment methods were deemed difficult to implement on the large-scale sponge funnel, therefore it was not explored in this study. Nanoparticle detachment and wash out would lead to a decrease in sorbent material properties over time [30], and would contaminate the aquatic environment and extracted oil. Therefore, the nanofiller was dispersed within the polymer matrix to minimize the potential of nanoparticle detachment in this study. The desired traits for a sorbent material used in the SOS: (i) facile fabrication of large-scale funnel-shaped geometries, (ii) rapid oil absorption (oleophilic), ( iii) high water contact angle (hydrophobic), (iv) durability and reusability, and (v) Joule heating capabilities for highly viscous oil absorption. The PCS mixture with various salt porogen:CB/PDMS prepolymer ratios and CB loadings were used to fabricate small and large sponge cubes for detailed material characterization to determine the optimal nanocomposite sorbent formulation for use in the SOS. First, the porosities of each sponge cube fabricated in this study were measured (Figure 2a,b). As expected, a larger amount of salt porogen in the PCS mixture increased the porosity of the sponge fabricated from 76.7 ± 0.4% for CB15P4 up to 89.3 ± 0.3% for CB15P9. Sponges with varying CB loadings did not have a significant difference in the porosity. However, scanning electron microscope (SEM) images shown in Figure 2 revealed that varying CB loadings did have a noticeable effect on the microstructure of the nanocomposite sponges.

**Figure 3:**
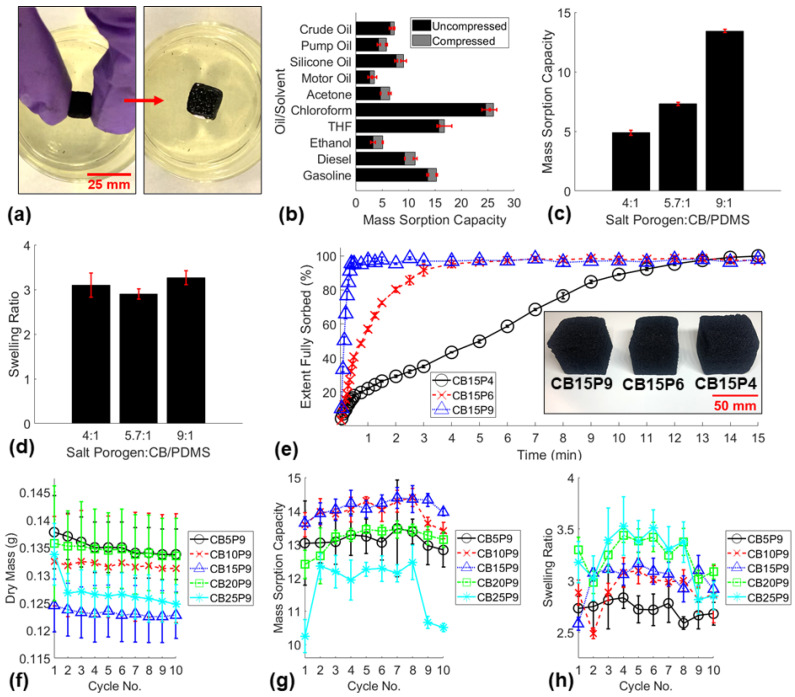
(**a**) Pictures of uncompressed gasoline sorption test before and after deploying a small CB15P9 sponge (mass sorption capacity = 13.6; swelling ratio = 3.3). (**b**) Mass sorption capacities of CB15P9 small sponges of various oils and organic solvents. (**c**) Mass sorption capacities, (**d**) swelling ratios, and (**e**) absorption rate comparison of varying porosity sponges. Durability study of most porous sponges with varying CB loadings including 10 cycles of (**f**) masses of dried sponges, (**g**) mass sorption capacities, (**h**) swelling ratios.

Next, the oleophilic properties and reusability of the fabricated sponges were characterized. The small CB15P9 sponge cubes were deployed in various oils and organic solvents at room temperature and allowed to fully sorb (Figure 3a). The mass sorption capacities of the sorbent material were measured, demonstrating the capability of absorbing a wide range of oils and organic solvents (Figure 3b). Notably, Figure 3b shows both uncompressed sorption capacities and compressed sorption capacities, which designate if the sponge was mechanically compressed while submerged to expel air bubbles that can become trapped during sorption. The trapped air may affect the oil flow rate through the sponge and consequently the extraction rate of the SOS. The mass sorption capacities and swelling ratios of the varying porosity small sponges deployed in gasoline were compared (Figure 3c,d). The mass sorption capacities were greater for higher porosity sponges, which agreed with similar studies in the field [31,32,33]. The absorption rates of the varying porosity large sponge cubes were compared by incrementally measuring the mass sorption capacities during 15 min of gasoline absorption (Figure 3e). The results showed that higher porosity sponges absorbed oil considerably more rapidly than lower porosity sponges. The highest porosity sponge (CB15P9) was found to fully sorb gasoline in under 1 min, while the lowest porosity sponge (CB15P4) required over 12 min to fully sorb. Therefore, PCS with the highest ratio (9:1) of salt porogen:CB/PDMS prepolymer was selected to fabricate sponges with varying CB loadings for the durability and reusability investigation.

A critical application of the SOS would be to replace single-use sorbent materials that are widely used in the oil spill cleanup industry that results in massive solid waste. To reduce or eliminate this waste, the sorbent material used in the SOS must be highly reusable. Herein, the durability and reusability of the nanocomposite sponges used in this study were investigated for 10 cycles of gasoline absorption, cleaning in ethanol, and drying in a vacuum oven. The average dry masses of the sponges before gasoline sorption, mass sorption capacities, and swelling ratios were measured for sponges with various CB loadings between 5–25 wt% (Figure 3f–h). A significant loss of mass was observed in CB5P9 sponges due to the lack of mechanical reinforcement in the polymer leading to significant damage, and the first cycle of CB25P9 sponges likely due to significant nanoparticle detachment from inadequate mixing of the high CB loading in the polymer matrix. After several cycles, noticeable damage was observed within the CB5P9, CB10P9, and CB25P9 sponges, while the CB15P9 and CB20P9 sponges remained entirely intact. Mechanical compression tests before and after 10 cycles of gasoline absorption and SEM images confirmed the apparent damage or lack of damage to the sponges (Figure S9). 

**Figure 4:**
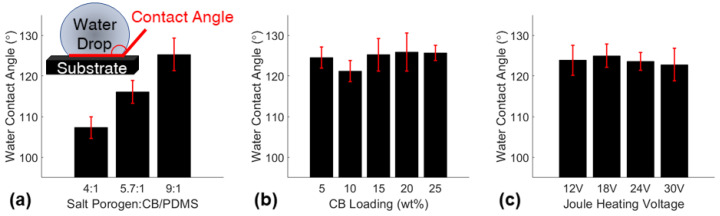
Water contact angles for (**a**) varying porosity sponges, (**b**) varying CB loading sponges, and (**c**) various Joule heating voltages applied to small CB20P9 sponges.

The hydrophobic properties of the sponges were investigated via the water contact angle test. The water contact angles comparison for the varying porosity sponges, varying CB loading sponges, and Joule heating voltages applied to a small CB20P9 sponge are shown in Figure 4 and representative images are shown in Figure S10. Higher porosity sponges displayed higher hydrophobicity with larger water contact angles which agreed with multiple studies in the field [31,34]. In contrast, CB loading did not influence the hydrophobic properties of the materials, which did not align with similar studies in the field that coat the surface of PDMS sponges with carbonaceous nanoparticles [35,36]. This finding and the SEM images of the nanostructure of the sponge surface (Figure S11) both confirmed that the CB is embedded within the polymer matrix. Lastly, the water contact angles for a CB20P9 sponge with various Joule heating voltages applied were confirmed to not affect the hydrophobicity of the sponge. Therefore, Joule heating may be implemented on the sponge without compromising the vital hydrophobic behavior of the sorbent. 

### 3.3. Sponge Joule Heating for Viscous Oil Absorption

**Figure 5:**
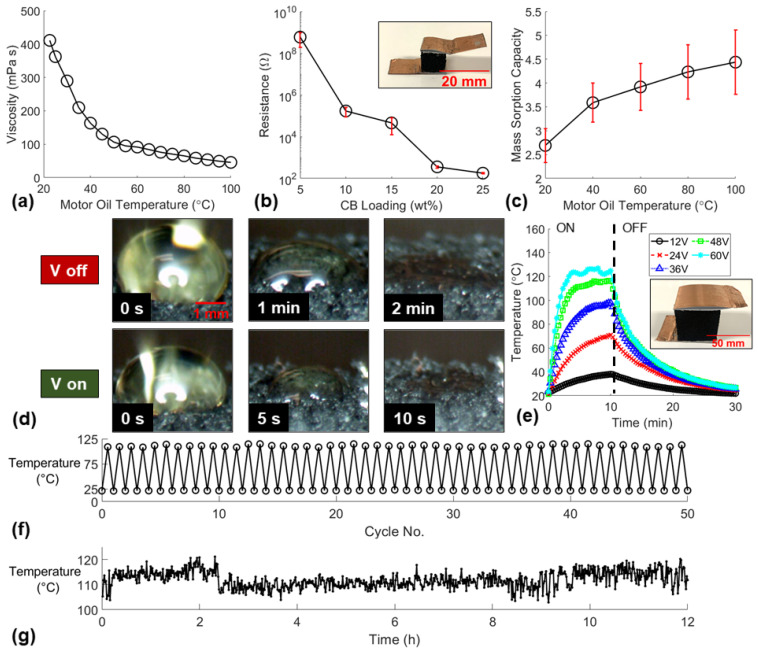
(**a**) Viscous motor oil change in viscosity due to change in oil temperature. (**b**) Resistances of small sponges with varying CB loadings (CB5P9, CB10P9, CB15P9, CB20P9, and CB25P9). (**c**) Mass sorption capacities of small CB20P9 sponges due to motor oil temperature change. (**d**) Microscopy images demonstrating CB20P9 sponges rapid absorption of viscous motor oil due to Joule heating. (**e**) Joule heating temperatures of a large CB20P9 sponge at various applied voltages. Joule heating durability study including (**f**) 50 cycles of maximum and minimum temperatures and (**g**) 12 h of continuous 30 V applied to a small CB20P9 sponge.

Highly viscous oil spills are exceedingly difficult to remove from water with sorbent materials as sorbents typically require a low viscosity fluid to readily absorb through the microstructure of the material. Many researchers have recently investigated the heating capabilities of nanocomposite sorbent materials via simulated sunlight (photothermal effect) [37] and Joule heating [38] to increase the local temperature and reduce the viscosity and rapidly absorb the nearby oil. Motor oil was the most viscous oil obtained and the viscosity was measured as oil temperature increased to confirm the significant decrease in viscosity (Figure 5a). Joule heating functionality was enabled when a sufficient CB loading was dispersed within the polymer matrix.

The semi-conductive behavior of the nanocomposite sponge with varying CB loadings used in this study was characterized by measuring the resistances of the small sponges (Figure 5b). The sponges with CB loadings between 5–15 wt% did not demonstrate Joule heating functionality for the voltages investigated. Additionally, CB25P9 sponges proved difficult to scale up due to the inadequate hand mixing process of the highly viscous nanocomposite prepolymer with the salt porogen (Figure S12). Therefore, CB20P9 sponges were selected as the ideal nanocomposite sorbent material formulation to explore Joule heating capabilities.

The mass sorption capacities of the small CB20P9 sponges were measured for motor oil at various temperatures to verify the sorbent absorbed the oil more readily at higher temperatures (Figure 5c). A microscopy experiment was conducted with no voltage applied and 30 V applied to a small CB20P9 sponge to compare the time required for the full absorption of a motor oil droplet (Figure 5d). The experiment proved that Joule heating offered over an order of magnitude improvement in the time required to absorb the viscous motor oil droplet if the sponge was Joule heated to temperatures near 100 °C (Figure S12). A large CB20P9 sponge was Joule heated with various voltages to investigate the heating rate of larger sorbents (Figure 5e). Higher voltages resulted in faster heating rates and higher maximum sorbent temperatures that reached 38 °C, 71 °C, 98 °C, 117 °C and 127 °C for 12 V, 24 V, 36 V, 48 V, and 60 V, respectively. The longevity of the Joule heating functionality was investigated for reusability by Joule heating the sponge for 50 cycles (Figure 5f), and endurance by continuously heating the sponge for 12 h (Figure 5g). The maximum and minimum temperatures over 50 cycles remained consistent at 111 ± 2 °C and 22.1 ± 0.5 °C, respectively, which demonstrated the essential reusability of the Joule heating functionality. Additionally, the endurance experiment proved the sorbent can maintain a steady temperature of 112 ± 3.2 °C over 12 h of constant Joule heating. These experiments proved the ability of the nanocomposite sorbent material used in the SOS to be Joule heated for consistent and reliable rapid absorption of viscous oils. While Joule heating the sponge funnel in the SOS was not within the scope of this study, this sorbent heating functionality may be utilized on the SOS for continuous extraction of highly viscous oils from water.

### 3.4. Oil Spill Cleanup Demonstrations

**Figure 6:**
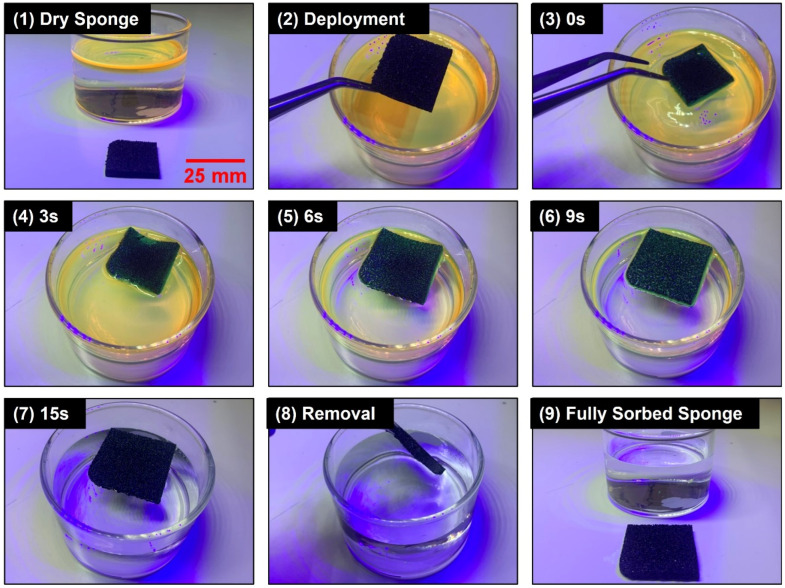
Images over time demonstrating the oil/water separation capability of a CB15P9 sponge.

After characterizing the oleophilic, hydrophobic, durability, reusability, and Joule heating properties of the nanocomposite sponge, it was necessary to test the viability of the material to absorb oil spilled in water. First, a sheet of nanocomposite sponge was deployed in a small-scale simulated gasoline spill in water (Figure 6). The sponge rapidly expanded and absorbed the small oil spill in seconds and was easily removed. This experiment demonstrated the oil/water separation capabilities of the sponge material. Many studies in this field demonstrate similar lab-scale oil/water separation capabilities of their sorbent material [39,40,41,42]. However, very few explore solutions to scale up this technology beyond this experiment, a step that is vital to real-world oil spill cleanup applications.

**Figure 7:**
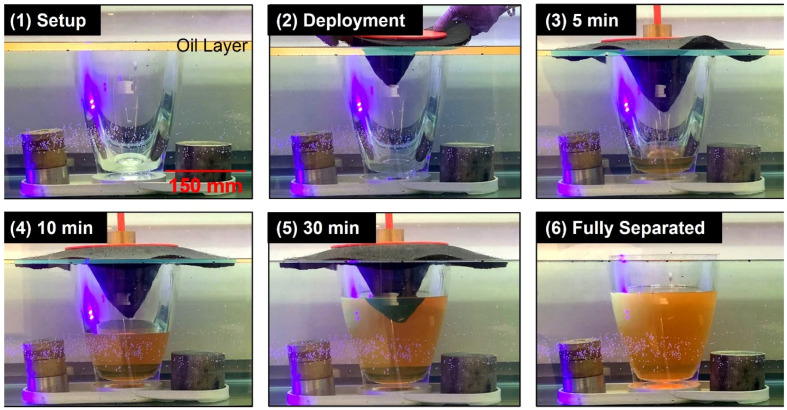
Images over time of a sponge funnel demonstrating gravity-driven oil/water separation to absorb oil from the water surface and fill a vessel.

A demonstration of the sponge funnel functionality was conducted to visualize the gravity-driven oil/water separation mechanism (Figure 7). A transparent vessel was submerged in a simulated gasoline spill in water such that the oil layer height was near the level of the top rim of the clear vessel. The sponge funnel, SOS lid and vent component, and a small weight were deployed on the clear vessel. The sponge funnel rapidly expanded while absorbing gasoline, and in about 5 min the sponge funnel was sorbed and began releasing gasoline from the hollow core of the sponge into the clear vessel in a steady and constant stream (Figure S13). This process continued for about 30 min until the entire oil spill was separated from the water surface into the vessel. Notably, the oil layer was just below the brim of the vessel to begin the experiment. Therefore, the sorbent material demonstrated the ability to pull the gasoline through the microstructure of the sponge and above the brim of the vessel via capillary action, owing to the impressive oleophilic properties of the sponge material. This visually proved the oil/water separation mechanism of the sponge funnel deployed in a vertically aligned vessel, demonstrating the feasibility of the SOS design.

**Figure 8:**
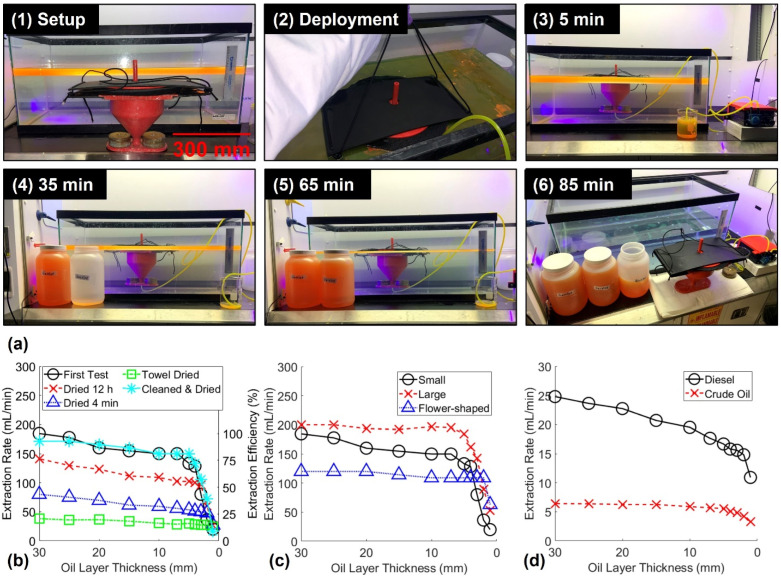
(**a**) Pictures over time of SOS continuous extraction of oil from water. (**b**) The SOS extraction rate for gasoline at various layer thicknesses for various sorbent reuse conditions and (**c**) for varying sponge funnel designs. (**d**) The SOS extraction rate of diesel fuel and crude oil at various oil layer thicknesses.

Lastly, the fully assembled floating SOS system was deployed in simulated oil spills of 3 gallons of gasoline to determine the extraction rate at various oil layer thicknesses (Figure 8a). The SOS was easily deployed via rope handles into the oil spill, the peristaltic pump connected underneath the floating vessel was turned on, and continuous oil extraction began within 5 min. In under 90 min, the SOS had extracted the 30 mm thick oil spill from the water. Notably, no water was observed in the extracted oil. This observation indicated that the SOS likely demonstrated an oil recovery efficiency (volume ratio of oil removed to the total fluid removed) near 100%. This apparent water-free oil extraction capability is remarkable and unique to the SOS as a vast majority of traditional oil skimmers report a significantly lower recovery efficiency [43]. The following experiments were aimed at testing the extraction rate of the SOS at different sponge funnel reuse conditions, various designs of the sponge funnel, and for various types of oil spilled on water.

The SOS containing the small diameter sheet sponge funnel was tested at various reuse conditions to test the extraction rate and efficiency at each oil layer thickness (Figure 8b). Each test demonstrated the maximum extraction rate at the thickest oil layer (30 mm) likely due to gravity increasing the rate of absorption on the top surface of the sponge sheet submerged in the thick oil layer. As the oil layer became thinner during extraction, the SOS progressively decreased in oil extraction rate and efficiency, as this is a common issue faced by oil skimmers [4]. This was likely attributable to the reduction of gravitational influence and sponge surface area in contact with the thin oil layer. The first test and the thoroughly cleaned and dried sponge funnel demonstrated the highest efficiency and extraction rate throughout the tests, which demonstrated the reusability of the entire SOS system. When the sponge funnel had not been adequately cleaned with ethanol and dried completely, the extraction efficiency decreased noticeably. For each reuse condition investigated, the sponge funnel had undergone previous expansion of the polymer such that significant air pockets formed within the microstructure of the sponge when removed from the oil spill and exposed to the atmosphere. The three mechanisms that formed these air pockets in the sponge funnel: (i) evaporation, (ii) gravity-driven oil separation from the sponge funnel, or (iii) compression and paper towel absorption. The mass sorption capacities of the large CB15P9 sponges were measured at each of the reuse conditions tested. The results showed that the general trend of the mass sorption capacities matched the extraction rate trend for the various sponge reuse conditions (Figure S14). Therefore, when more air pockets were trapped within the sponge funnel (lower mass sorption capacity) there was a significant loss in flow rate through the microstructure of the sponge that reduced the SOS extraction rate.

The purpose of the sponge sheet in the sponge funnel design was to introduce a sorbent with a high surface area in contact with the spilled oil for rapid extraction. To explore the potential of increasing the surface area of the sponge sheet to increase the extraction rate, a sponge funnel with a larger diameter and one with a flower-shaped design were fabricated and tested (Figure 8c). As expected, the larger the surface area of the circular sponge sheet demonstrated a faster extraction rate than the smaller circular sponge sheet sponge funnel. Surprisingly, the flower-shaped design performed the worst despite having the largest total surface area in contact with the oil layer. This was likely due to a significant reduction in gravitational influence as the flower-shaped sponge sheet floated on the oil layer more readily and the top surface area in contact with the oil was reduced with this design. However, the flower-shaped sponge funnel retained its extraction efficiency for very thin oil layers compared to the circular sheets, likely due to increased surface area in contact with the thin oil sheens. Lastly, the large-diameter circular sponge funnel was tested in higher viscosity diesel fuel and crude oil simulated spills (Figure 8d). While the SOS was able to continuously extract the higher viscosity oils, the extraction rate was significantly reduced showing the potential to improve the SOS design by incorporating Joule heating.

## 4. Conclusions

In conclusion, a novel energy-efficient oil skimmer was developed and tested. For the first time, a highly reusable, floating vessel-type oil collecting device was constructed with continuous oil extraction capabilities. The parameters of the facile sorbent fabrication method including the amount of porogen and CB loadings were investigated to determine the ideal nanocomposite sponge formulations. The desired characteristics of the sponge included a rapid oil absorption rate, sufficient hydrophobicity, durability for reuse, and Joule heating for viscous oil absorption. The ideal sponge types for use in the SOS were determined to be CB15P9 for low viscosity oil spills and CB20P9 to incorporate Joule heating functionality for viscous oil absorption. The SOS device demonstrated great reusability, a high oil extraction rate, and the ability to extract multiple types of oils. The SOS is a promising new oil spill cleanup technology that may help solve the problem of massive amounts of solid waste produced by single-use sorbents during oil spill cleanup, in addition to other applicable large-scale oil skimming applications.

## Data Availability

Not applicable.

## Supplementary Materials

The following supporting information can be downloaded at: https://www.mdpi.com/article/10.3390/polym14112269/s1, Figure S1: Images of the preliminary investigation of spherical shell-structured PDMS sponge sorbents including the (a,b) the fabrication method of covering the salt ball with PCS, (c,d) a fabricated hollow spherical sponge, and (e) the scalability of the salt ball porogen; Figure S2: Images of the preliminary spherical hollow shell sponge oil absorption in water experiment including (a) deployment, (b) oil absorption and separation from water, (c) extraction of the encased oil inside the shell sponge, and (d) depositing extracted oil into a separate container; Figure S3: Images of (a) fabricated spherical shell sponge with attached connector for oil extraction, (b) deployment of the hollow sponge device in a simulated oil spill, (c) pumping to remove oil, and (d) proof of oil extraction from water; Figure S4: (a) The design of the nanocomposite sponge funnel, (b) the steps to fabricate the sponge funnel, and (c) pictures of a fabricated sponge funnel; Figure S5: (a) Design of the first SOS prototype and (b) the labeled cross-section including a ballast tank to control the buoyancy. Pictures of the fabricated SOS prototype with a ballast tank (c) before and (d) after deployment in a simulated oil spill; Figure S6: (a) Pictures of the least porous sponge (top) and the most porous sponge (bottom) under compression from a 120 g book. (b) Pictures of a small cube sponge uncompressed (left) and compressed to 80% compressive strain. (c) Stress-strain curves of the varying porosity sponges. (d–h) Stress-strain curves of varying CB loading sponges before and after 10 cycles of gasoline sorption and SEM images of the sponge microstructure after 10 cycles. (i) Representative stress-strain curves of 500 cycles of CB15P9 submerged in gasoline; Figure S7: (a) Schematic showing the water contact angle of a droplet on a substrate. Representative light microscopy images of a water droplet deposited on the (b–d) varying porosity sponges, (e–h) varying CB loading sponges, and (i–l) varying Joule heating voltages applied to a CB20P9 sponge; Figure S8: High magnification SEM images of the surface of a CB15P9 sponge that shows the CB embedded within the PDMS matrix; Figure S9: (a) Image of the Joule heating experimental setup and measured temperature change due to 3 min of various voltages applied to a (b) CB20P9 and (c) CB25P9 small cube sponge. (d) Picture of the CB20P9 and CB25P9 large cube sponges showing the shrinkage of sponges fabricated with a 25 wt% CB loading due to the difficulty of mixing the viscous prepolymer with the salt porogen; Figure S10: Image of the nanocomposite sponge funnel demonstrating the gravity-driven oil/water separation capabilities in a simulated gasoline spill in water; Figure S11: Mass sorption capacities of large CB15P9 sponges in gasoline with the same sponge reuse conditions as the sponge funnel reuse conditions tested in the SOS gasoline extraction from water experiments; Figure S12: Images of the PCS templating fabrication methods used (a) to fabricate sponge cubes, (b) a circular sponge sheet, and (c,d) the flower-shaped sponge funnel with 60 pedals; Figure S13: Images of the (a) small cube sponges and tools and materials used to attach electrodes and (b) the multimeter measuring the resistance of a small cube sponge; Figure S14: Images of the large salt porogen fabrication method including (a) molding the cylindrical base and (b) the cone top, (c) stacking the cone onto the cylindrical base, and (d) hardening the extended cone-shaped porogen in a commercial microwave; Table S1: Designation of Sponge Materials Investigated; Video S1: Demonstration of oil cleaning process.
