# Supplementary material for: Funnel-Shaped Floating Vessel Oil Skimmer with Joule Heating Sorption Functionality
Source: Polymers (Basel). 2022 Jun 2;14(11):2269. doi: 10.3390/polym14112269 (PMC9182707; doi:10.3390/polym14112269)
Supplement: Supplementary file 1 [file polymers-14-02269-s001.zip › polymers-1721531-supplementary.pdf]

Supporting Information

Funnel-Shaped Floating Vessel Oil Skimmer with Joule Heating Sorption Functionality

Blake Herren, Mrinal C. Saha, M. Cengiz Altan and Yingtao Liu *

School of Aerospace and Mechanical Engineering, University of Oklahoma, Norman, OK 73019, USA; blake.herren@ou.edu (B.H.); msaha@ou.edu (M.C.S.); altan@ou.edu (M.C.A.)
* Correspondence: yingtao@ou.edu; Tel.: +1-(405)-325-3663

Table S1. Designation of Sponge Materials Investigated.

Sponge Material Designation	Salt Porogen: CB/PDMS Prepolymer	CB wt% in CB/PDMS Prepolymer
CB15P4	4:1	15
CB15P6	5.7:1	15
CB15P9	9:1	15
CB5P9	9:1	5
CB10P9	9:1	10
CB20P9	9:1	20
CB25P9	9:1	25

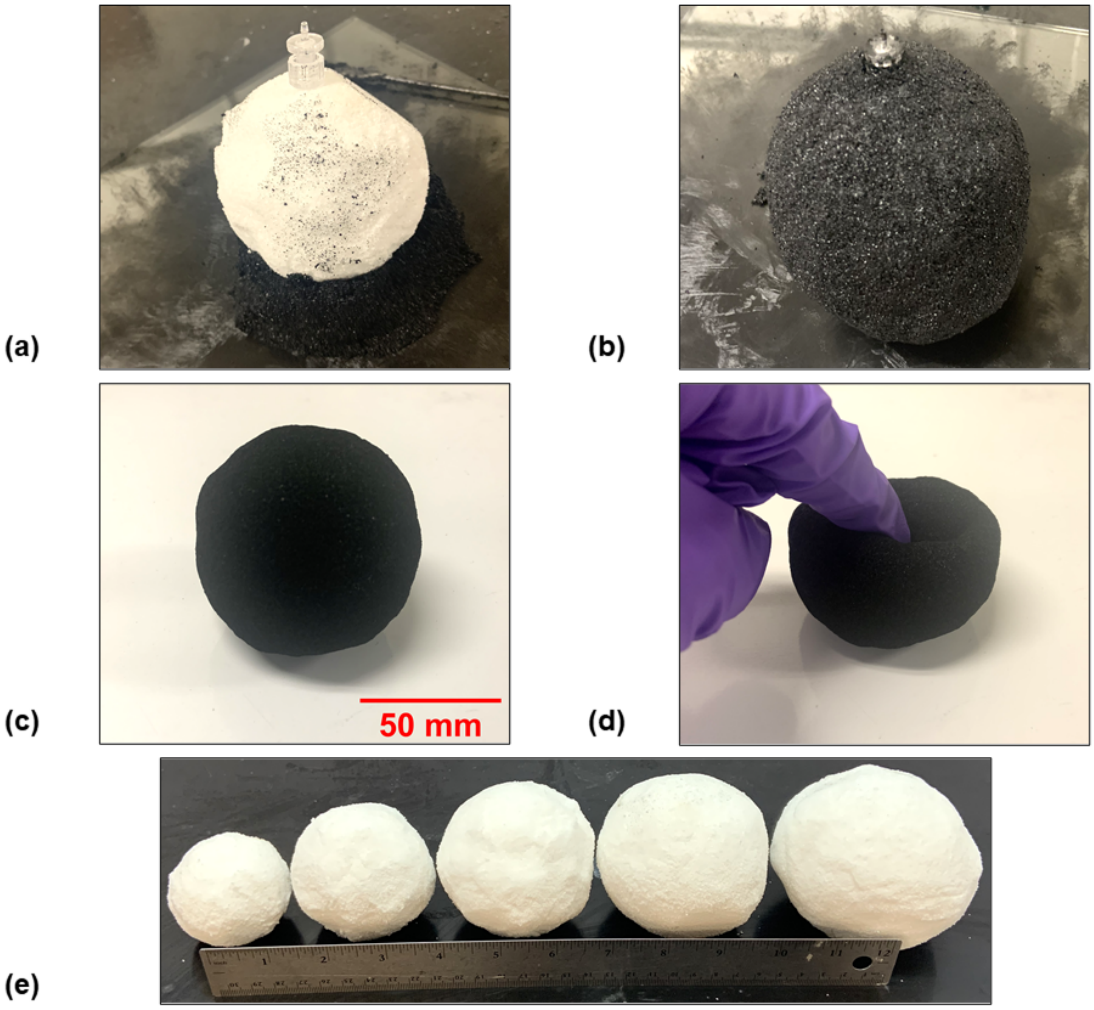

Figure S1. Images of the preliminary investigation of spherical shell-structured PDMS sponge sorbents including the (**a**,**b**) the fabrication method of covering the salt ball with PCS, (**c**,**d**) a fabricated hollow spherical sponge, and (**e**) the scalability of the salt ball porogen.

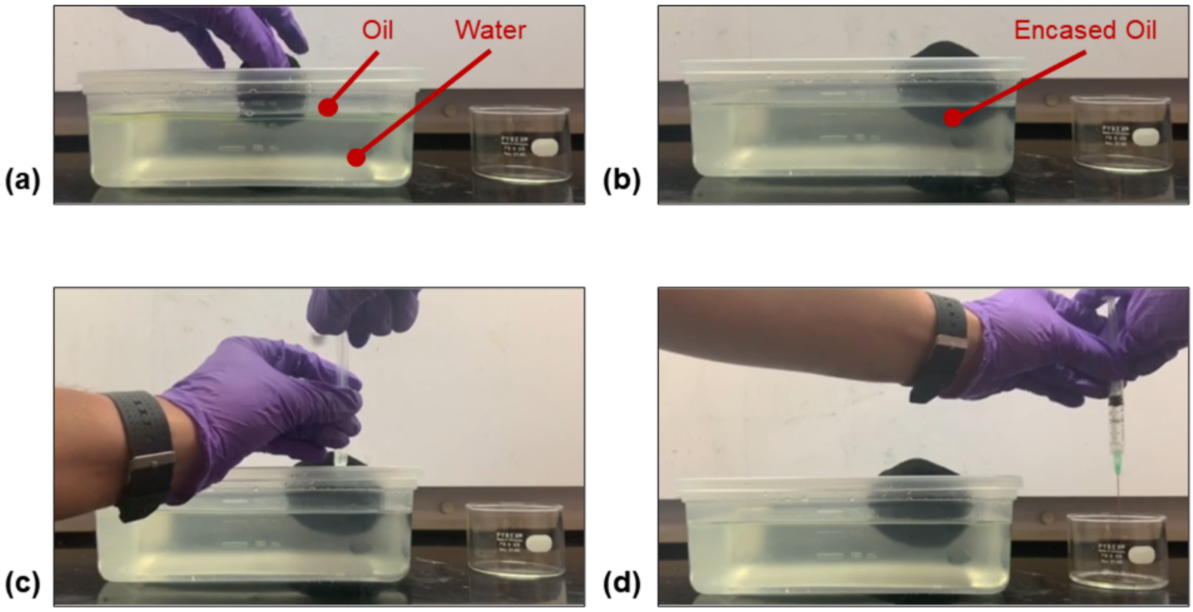

Figure S2. Images of the preliminary spherical hollow shell sponge oil absorption in water experiment including (**a**) deployment, (**b**) oil absorption and separation from water, (**c**) extraction of the encased oil inside the shell sponge, and (**d**) depositing extracted oil into a separate container.

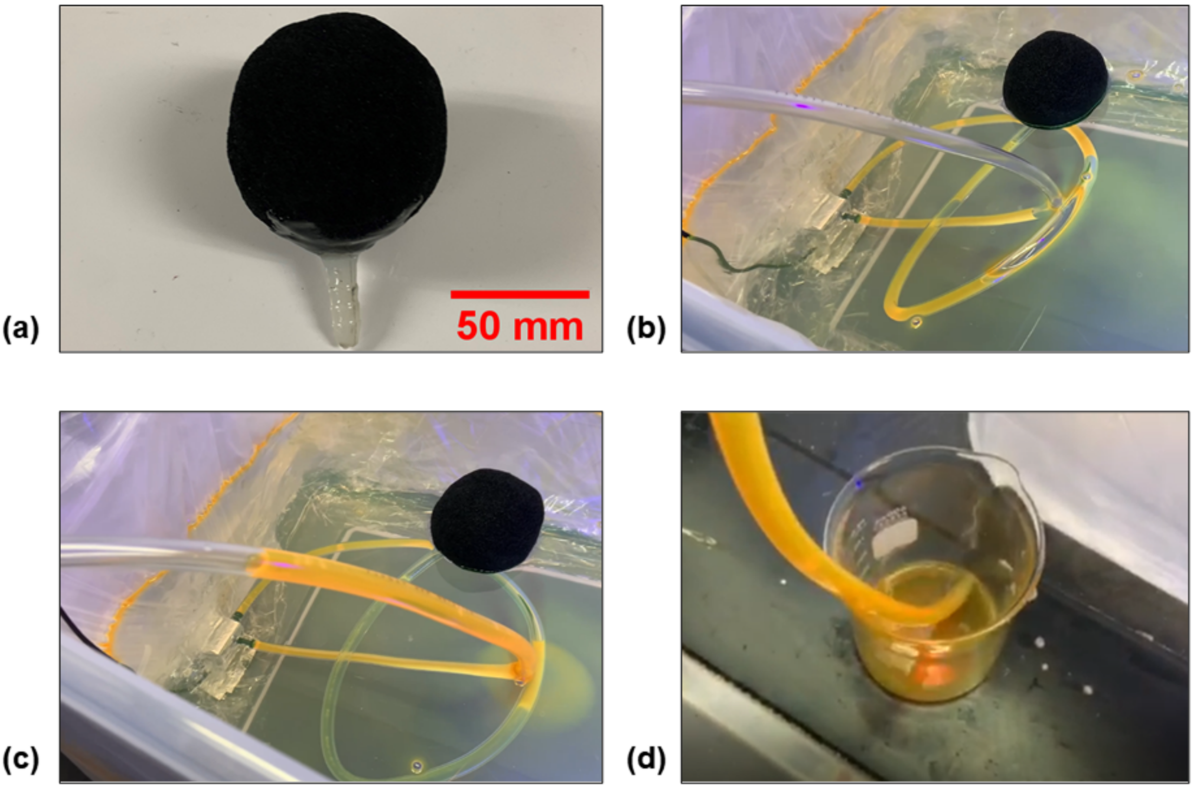

Figure S3. Images of (**a**) fabricated spherical shell sponge with attached connector for oil extraction, (**b**) deployment of the hollow sponge device in a simulated oil spill, (**c**) pumping to remove oil, and (**d**) proof of oil extraction from water.

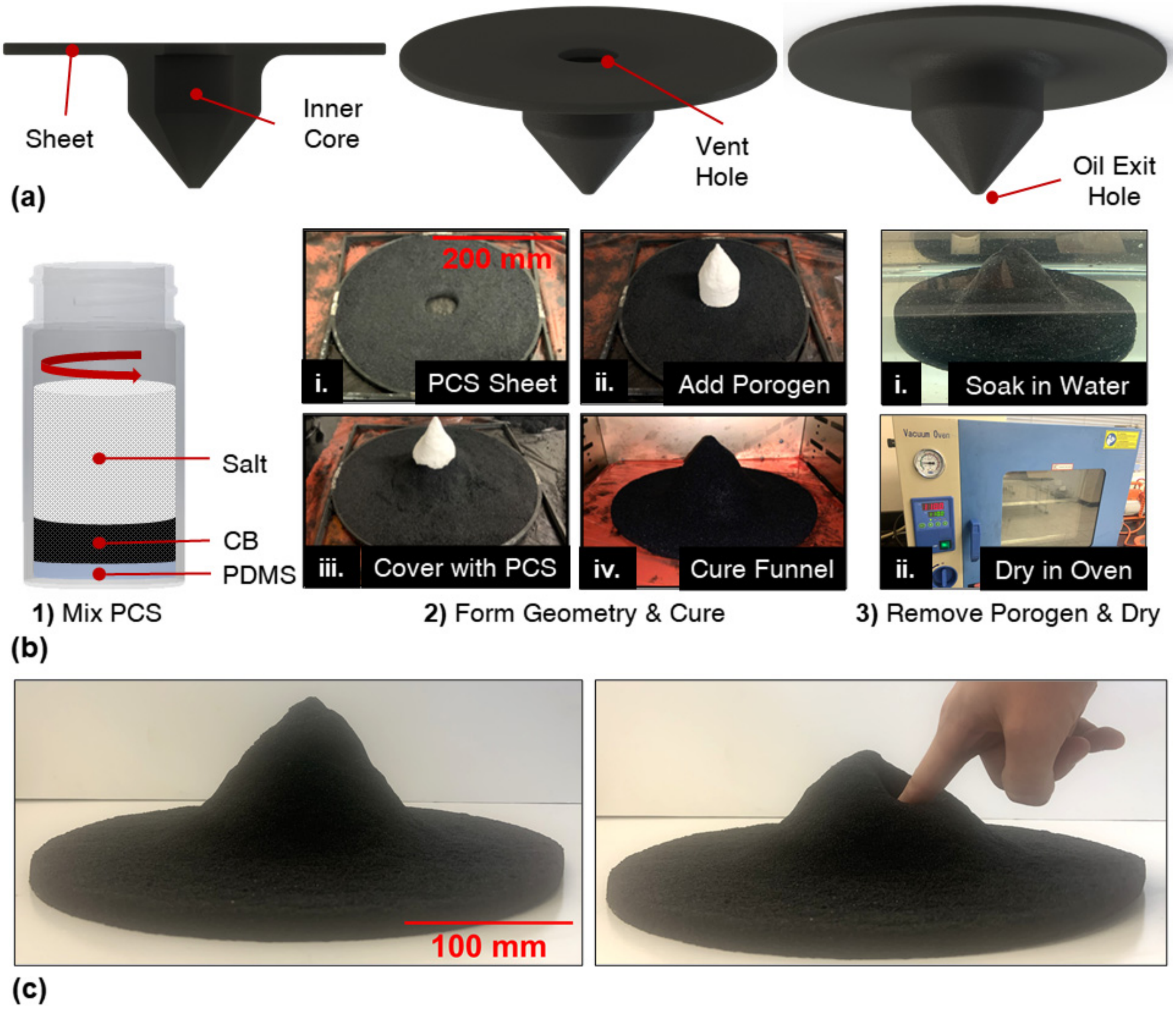

Figure S4. (**a**) The design of the nanocomposite sponge funnel, (**b**) the steps to fabricate the sponge funnel, and (**c**) pictures of a fabricated sponge funnel.

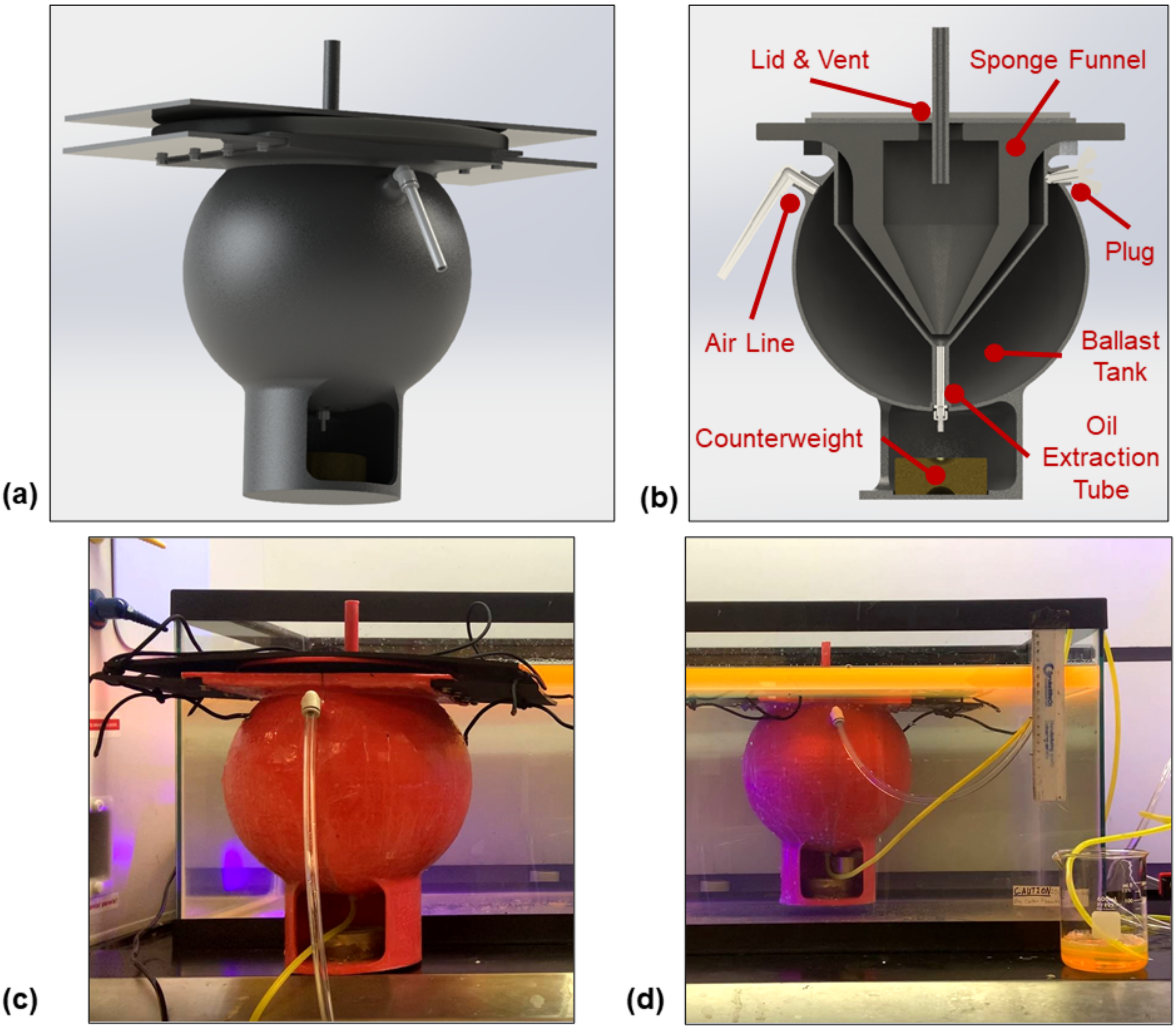

Figure S5. (**a**) Design of the first SOS prototype and (**b**) the labeled cross-section including a ballast tank to control the buoyancy. Pictures of the fabricated SOS prototype with a ballast tank (**c**) before and (**d**) after deployment in a simulated oil spill.

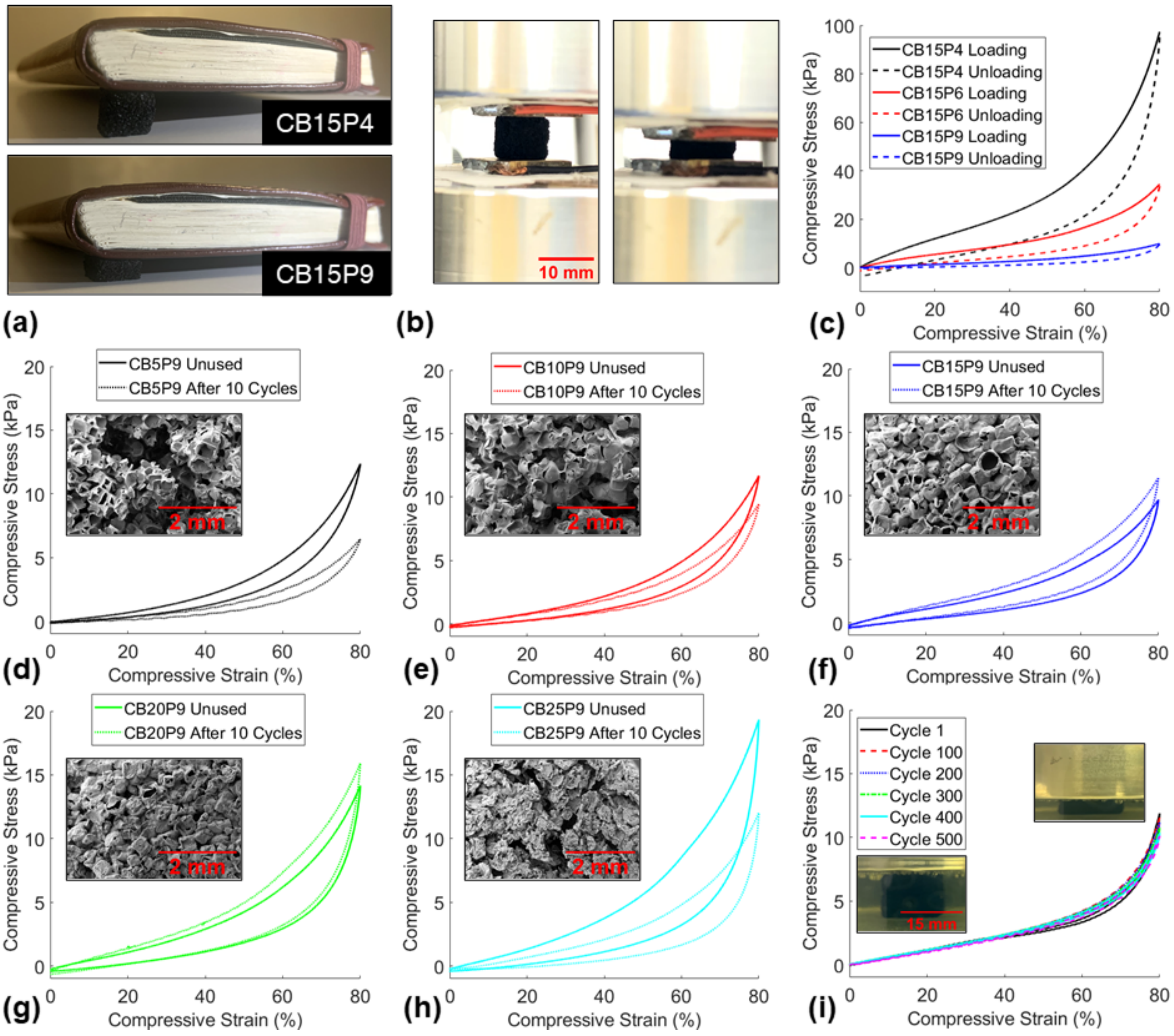

Figure S6. (**a**) Pictures of the least porous sponge (top) and the most porous sponge (bottom) under compression from a 120 g book. (**b**) Pictures of a small cube sponge uncompressed (left) and compressed to 80 % compressive strain. (**c**) Stress-strain curves of the varying porosity sponges. (**d**–**h**) Stress-strain curves of varying CB loading sponges before and after 10 cycles of gasoline sorption and SEM images of the sponge microstructure after 10 cycles. (**i**) Representative stress-strain curves of 500 cycles of CB15P9 submerged in gasoline.

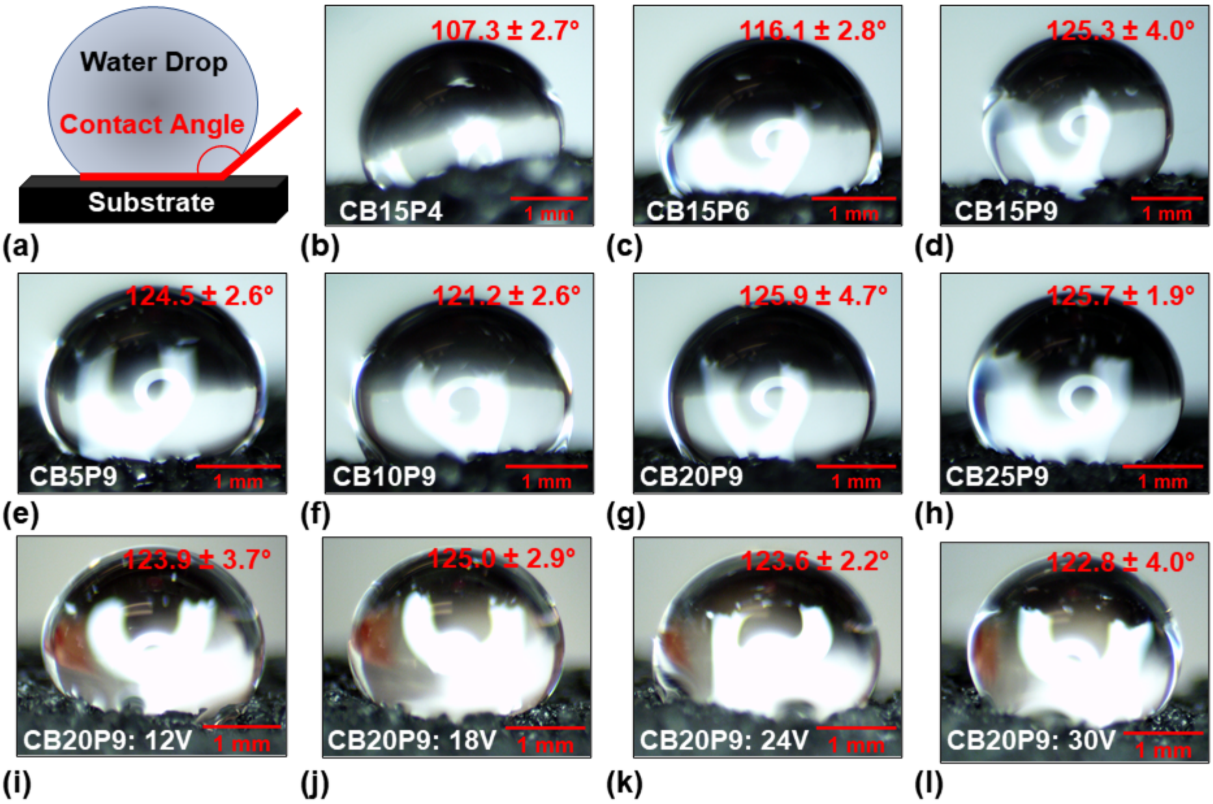

Figure S7. (**a**) Schematic showing the water contact angle of a droplet on a substrate. Representative light microscopy images of a water droplet deposited on the (**b**–**d**) varying porosity sponges, (**e**–**h**) varying CB loading sponges, and (**i**–**l**) varying Joule heating voltages applied to a CB20P9 sponge.

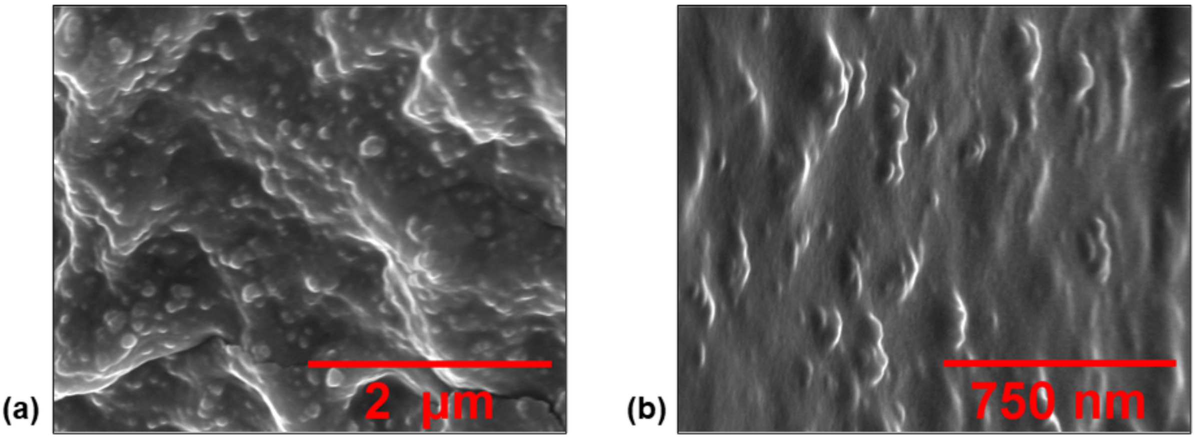

Figure S8. High magnification SEM images of the surface of a CB15P9 sponge that shows the CB embedded within the PDMS matrix.

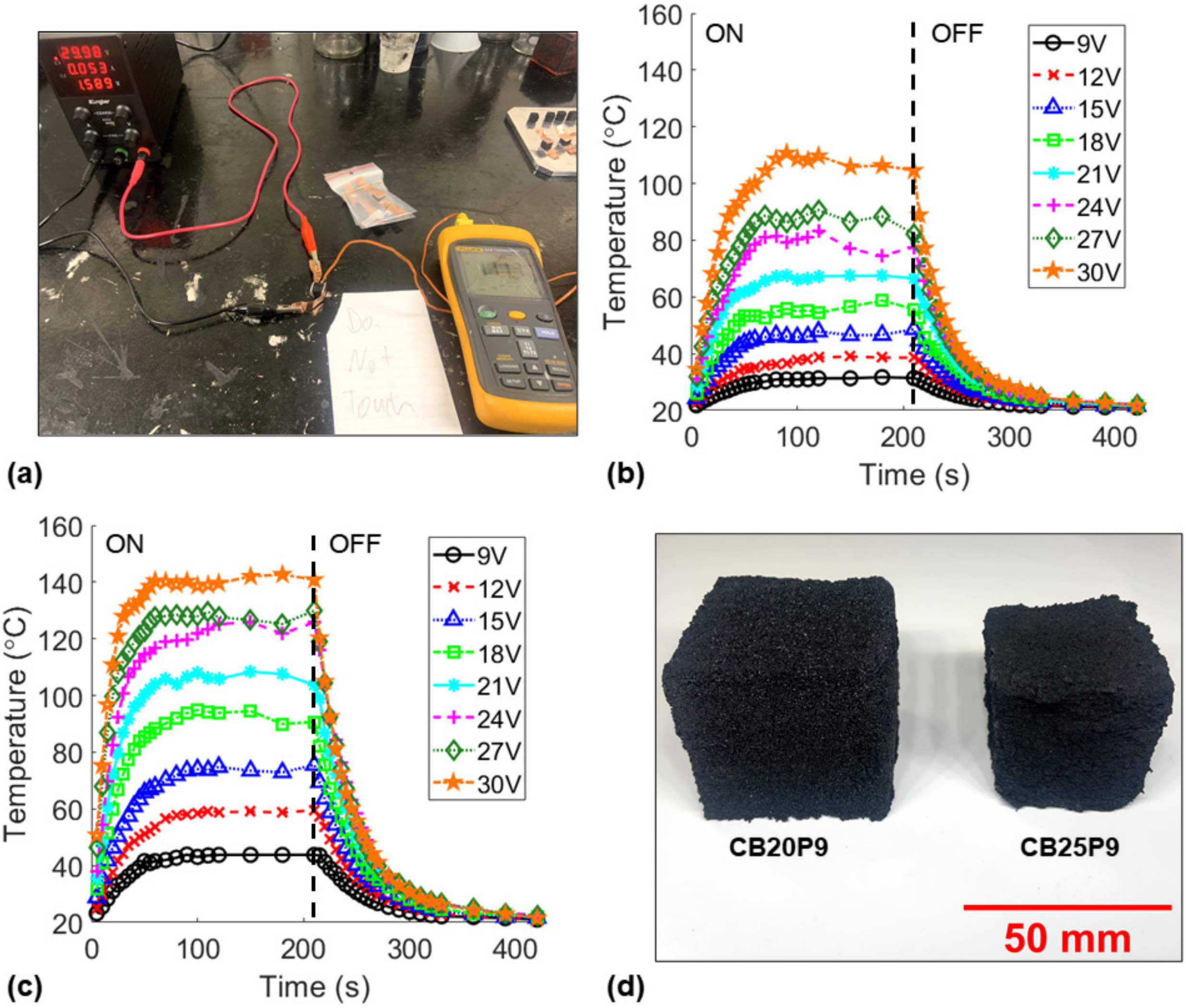

Figure S9. (**a**) Image of the Joule heating experimental setup and measured temperature change due to 3 min of various voltages applied to a (**b**) CB20P9 and (**c**) CB25P9 small cube sponge. (**d**) Picture of the CB20P9 and CB25P9 large cube sponges showing the shrinkage of sponges fabricated with a 25 wt% CB loading due to the difficulty of mixing the viscous prepolymer with the salt porogen.

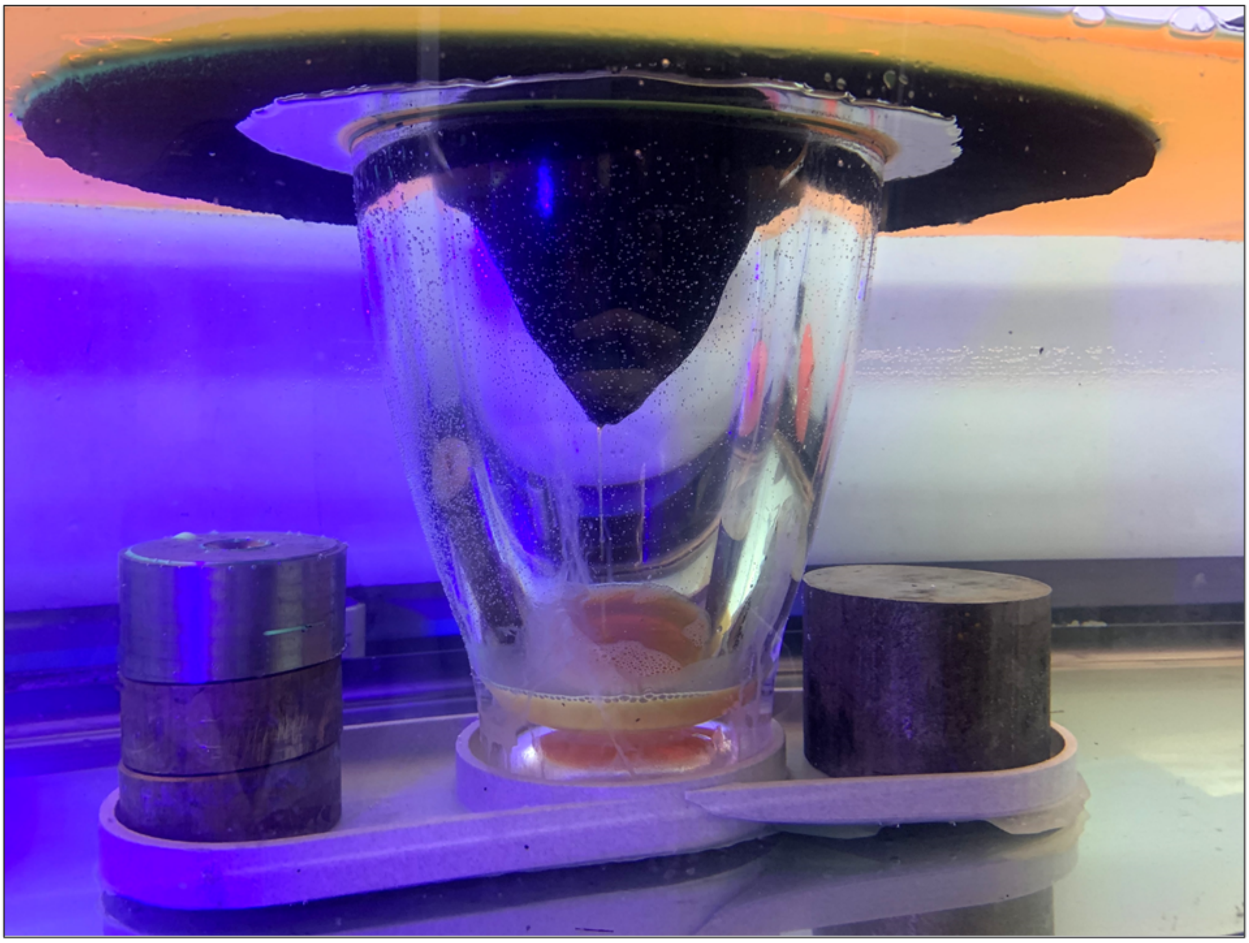

Figure S10. Image of the nanocomposite sponge funnel demonstrating the gravity-driven oil/water separation capabilities in a simulated gasoline spill in water.

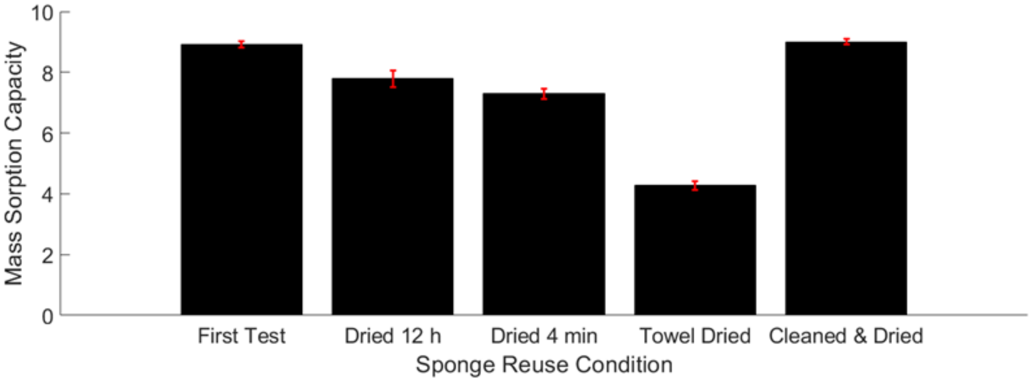

Figure S11. Mass sorption capacities of large CB15P9 sponges in gasoline with the same sponge reuse conditions as the sponge funnel reuse conditions tested in the SOS gasoline extraction from water experiments. .

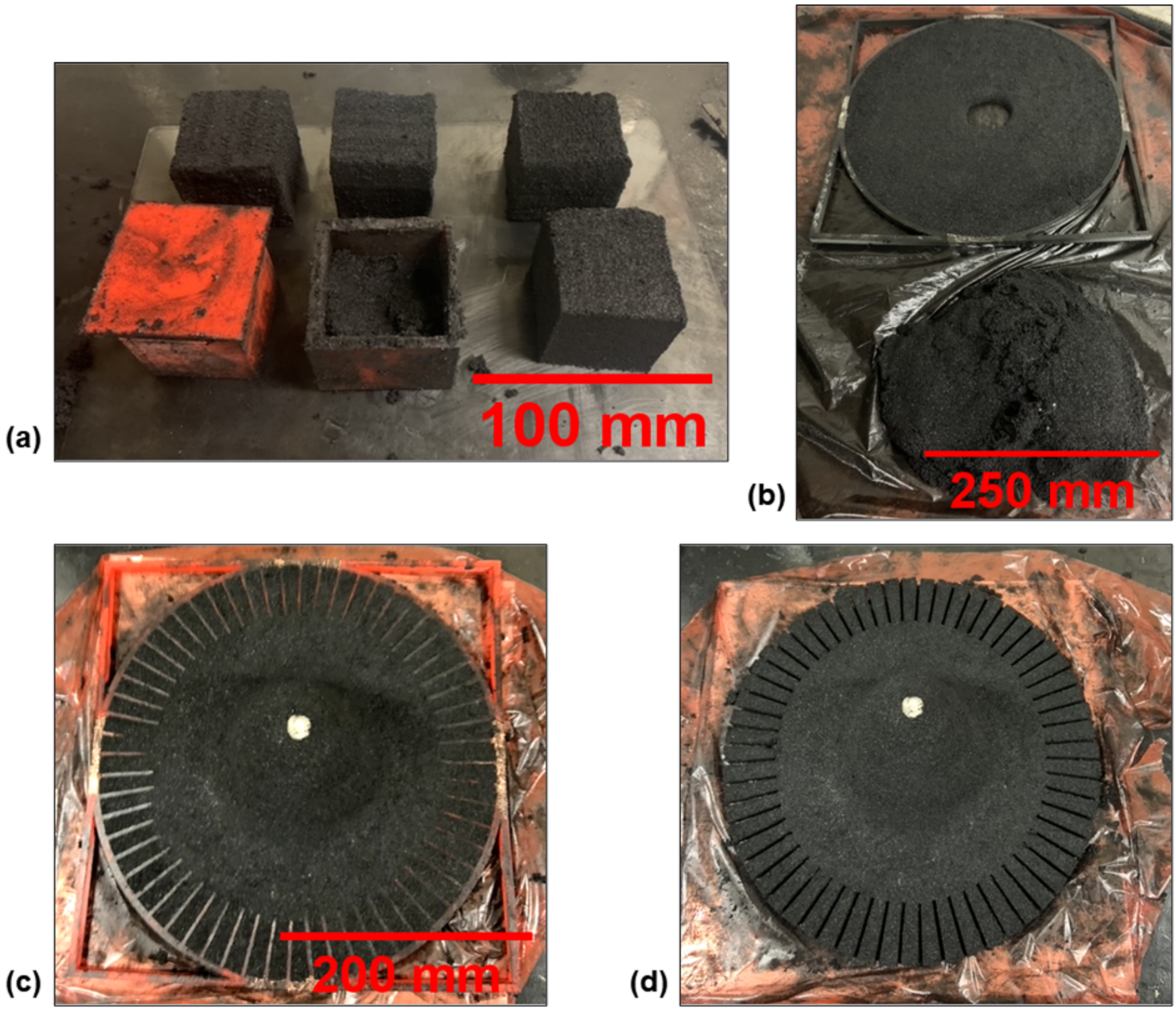

Figure S12. Images of the PCS templating fabrication methods used a) to fabricate sponge cubes, b) a circular sponge sheet, and c,d) the flower-shaped sponge funnel with 60 pedals.

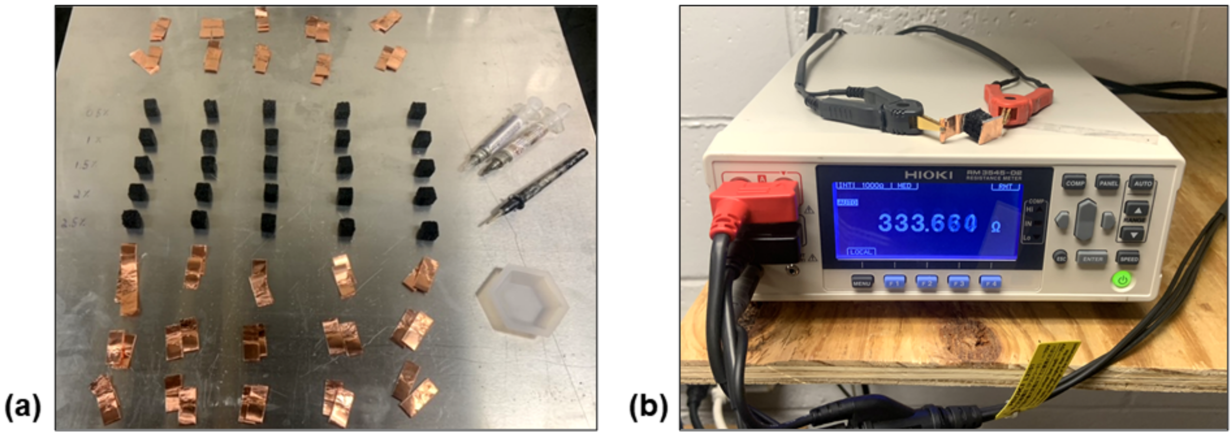

Figure S13. Images of the (**a**) small cube sponges and tools and materials used to attach electrodes and (**b**) the multimeter measuring the resistance of a small cube sponge.

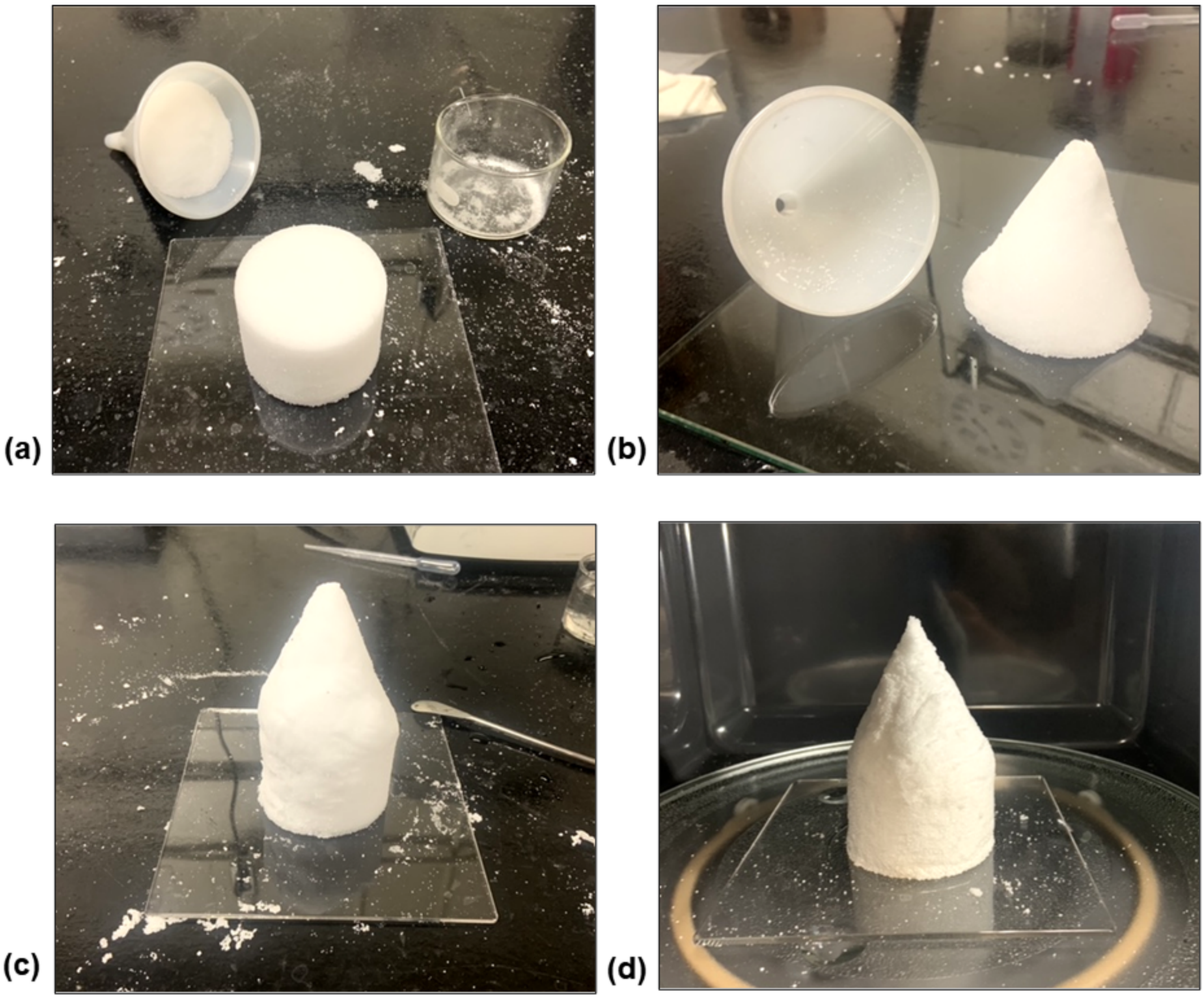

Figure S14. Images of the large salt porogen fabrication method including (**a**) molding the cylindrical base and (**b**) the cone top, (**c**) stacking the cone onto the cylindrical base, and (**d**) hardening the extended cone-shaped porogen in a commercial microwave.
